# Retrospective review of the use and costs of routine chest x rays in a trauma setting

**DOI:** 10.1186/1752-2897-7-2

**Published:** 2013-05-09

**Authors:** Kristina Ziegler, James M Feeney, Colleen Desai, David Sharpio, Wiiliam T Marshall, Michael Twohig

**Affiliations:** 1 Departments of Surgery, Stamford Hospital, 30 Shelburne Road, Stamford, CT 06904, USA; 2 Department of Surgery, Division of Trauma, Saint Francis Hospital and Medical Center, 114 Woodland St., Hartford, CT 06103, USA; 3 School of Medicine, University of Connecticut, 263 Farmington Ave., Farmington, CT 06032, USA

## Abstract

**Introduction:**

Chest x-rays (CXR) are routinely obtained on blunt trauma patients. Many patients also receive additional imaging with thoracic computed tomography scans for other indications. We hypothesized that in hemodynamically normal, awake and alert blunt trauma patients, CXR can be deferred in those who will also receive a TCT with significant cost savings.

**Methods:**

We retrospectively reviewed the charts of trauma patients from 1/1/2010 to 12/31/2010 who received both a CXR and TCT in the trauma room. Billing and cost data were collected from various hospital sources.

**Results:**

239 patients who met inclusion and exclusion criteria and received CXR and TCT between 1/1/2010 and 12/31/2010. The sensitivity of CXR was 19% (95% CI: 10.8% to 31%) and the specificity was 91.7% (95% CI: 86.7% to 95%). The false positive rate for CXR was 35.8% (95% CI: 21.7% to 52.8%) and the false negative rate was 24.5% (95% CI: 18.8% to 31.2%). The precision of CXR was 42.3% (95% CI: 25.5% to 61.1%) and the overall accuracy was 74.1% (95% CI: 68.1% to 79.2%). If routine chest xray were eliminated in these patients, the estimated cost savings ranged from $14,641 to $142,185, using three different methods of cost analysis.

**Conclusions:**

In patients who are hemodynamically normal and who will be receiving a TCT, deferring a CXR would result in an estimated cost savings up to $142,185. Additionally, TCT is more sensitive and specific than CXR in identifying injuries in patients who have sustained blunt trauma to the thorax.

## Introduction and Background

Trauma is the leading cause of death in young patients, and the fifth leading cause of death in those older than 65 [1]. In 2007 unintentional injuries accounted for 120,000 deaths and over 26 million disabling injuries in the United States with an estimated economic impact of $684.4 billion [1]. Major thoracic injuries are particularly devastating and are responsible for 20 to 25% of all trauma deaths [2]. Early identification of these injuries is important to make care decisions for trauma patients and to predict their outcomes. In severely injured patients, it is especially important to identify all injuries as quickly as possible. Lung contusions in particular contribute greatly to the morbidity and mortality of patients who have suffered thoracic trauma and are independent risk factors for the development of acute respiratory distress syndrome, pneumonia, and long-term respiratory dysfunction, with a mortality rate of 10% to 25% [3-6].

The Advanced Trauma Life Support guidelines of the American College of Surgeons include chest x-rays (CXR) as a routine adjunct to the primary trauma survey [7]. Previously, x-rays were also used regularly to evaluate the cervical spines of trauma patients. As imagining technology has evolved, computed tomography (CT) scans have largely replaced cervical spine x-rays in trauma patients [8]. There is also research demonstrating that pelvic x-rays show poor sensitivity and specificity for pelvic fractures, and that pelvic CT scans are superior imaging studies, and may safely replace plain pelvic x-ray in some circumstances, with a considerable resultant cost savings to the institution [9,10].

Similar to cervical spine and pelvic imaging, several studies suggest that CXR also has poor sensitivity and specificity in detecting thoracic injury in hemodynamically normal blunt trauma patients [2,3,11-17]. Instead, in these patients, it may be possible to use history and physical exam to determine whether or not imaging studies would alter patient management [18,19]. If imaging is warranted, thoracic computed tomography (TCT) scans demonstrate superior sensitivity and specificity compared to CXR in detecting a variety of thoracic injuries and may be a better study of choice [4,11,12,16,17,20].

At our institution blunt trauma patients routinely receive a supine CXR in the trauma room as an adjunct to the primary trauma survey regardless of history and physical exam findings. Many of these patients also receive CT imaging of the chest, abdomen, or pelvis. Whether or not a patient receives additional imaging depends on a variety of factors. These include a severe mechanism of injury, complaints of pain or other symptoms, concerning findings on physical exam, trauma surgeon preference, and other factors. We hypothesized that there would be significant cost savings at our institution if CXR were deferred in hemodynamically normal blunt patients who will proceed to CT for other indications.

## Methods

This project was approved by the Institutional Review Board (IRB) at Saint Francis Hospital and Medical Center (SFH), a Level II Trauma Center in Hartford, Connecticut, and by the IRB at the University of Connecticut School of Medicine in Farmington, Connecticut. After a query of the Trauma Database was performed the charts of all patients who received a CXR in one of the two trauma bays at Saint Francis between 1/1/2010 and 12/31/2010 were reviewed. Patients were included if they presented to the trauma room at our institution. Patients younger than 18 years old were excluded, as were patients with penetrating trauma or patients with any hemodynamic abnormality, clear respiratory failure requiring immediate intervention, needle decompression in the field, or obvious signs or symptoms of thoracic injury which were deemed at the time to need immediate intervention. In addition, patients were excluded if they had a Glasgow Coma Scale less than 14, were otherwise altered so as to render physical exam unreliable, if they only received CXR and no TCT, or had a completely negative physical exam with no indication for TCT but received one as part of a comprehensive scan. The radiology reports were reviewed in the medical record, as were history and physical exam findings at the time of admission. Images were reviewed on web based pictorial and archiving communications software (SECTRA PACS web V11.2).

The presence and types of each thoracic injury diagnosed on CXR were compared with those found on TCT, which was considered the gold standard. Any thoracic symptoms were noted, as were thoracic physical exam findings. Age and gender were reviewed for all included patients. Fisher's exact test was used to calculate p-values. Descriptive statistics and 95% confidence intervals (95% CI) were calculated.

The standard CT protocol for trauma in our institution consists of 120cc of Omnipaque 360 (GE Healthcare Inc, Princeton, NJ) with 5 mm cuts through the chest with 3mm reconstructions through the thoracic spine. All CT findings were reviewed, but only thoracic findings were noted for this study.

Methods for charge and cost analysis were used as published elsewhere [21]. Hospital costs were calculated using Activity Based Costing (ABC), a detailed analysis of direct, variable, and indirect costs associated with the performance of CXR, and using hospital charges as a surrogate for hospital costs. Medicare reimbursement for CXR was also determined.

## Results

**Figure 1:**
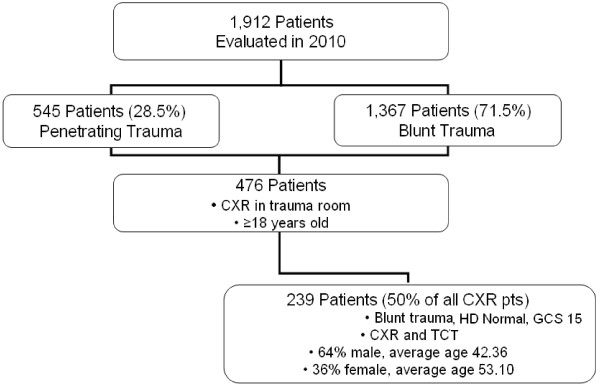
Patient Selection.

A total of 1,912 patients were evaluated in the two trauma rooms at SFH between 1/1/2010 and 12/31/2010, 1,307 of which were admitted. Those with blunt trauma comprised 1,367 (71.5%) patients and the remaining 545 (28.5%) sustained penetrating injuries. A total of 476 patients who received CXR in the trauma room met our initial inclusion criteria and of these, 239 (50% of included patients) were not excluded. These 239 patients received both CXR and TCT as part of their initial assessment (Figure 1). Of these patients, 64% were male with an average age of 42.4 years (39.14 to 45.58 years) and 36% were female with an average age of 53.1 years (47.8 to 58.5 years) (Table 1). All were hemodynamically normal at the time they proceeded to CT. The majority of patients received CT of the Chest/Abdomen/Pelvis for trauma. Each CXR was considered to be correct or incorrect, based on whether the CXR correctly identified all injuries noted on TCT.

**Table 1:** Patients included in study (n = 239)

	**Number and percent of patients**	**Average age**
		**(Years) (95% CI)**
**Male**	153 (64%)	42.36 (39.14 to 45.58)
**Female**	86 (36%)	53.10 (47.76 to 58.45)

The indications for TCT were as follows: subjective complaints of chest or back pain (167 patients; 69.9%, (95% CI 63.8% to 75.4%)), tenderness to palpation of the chest or back (69 patients; 28.9% (95% CI 23.5 to 34.9%), or widened mediastinum on CXR (4 patients; 1.7% (95% CI 0.5% to 4.4%)).

Of the 239 patients included, twenty one patients (8.8%) were found to have a pneumothorax, 3 of which were found on CXR (14.3% of pneumothoraces) (Table 2). Thirty three patients (13.8%) were found to have rib fractures, 13 of which were found on CXR (39.4% of rib fractures). Of the 15 lung contusions identified (6.3% of patients), 8 were discovered on CXR (53.3% of lung contusions). Fractures of the sternum were present in 5 patients (2.1%), one of which was found on CXR (20% of sternal fractures). The sensitivity of CXR was 19% (95% CI: 10.8% to 31%) and the specificity was 91.7% (95% CI: 86.7% to 95%) (Table 3). The false positive rate for CXR was 8.3% (95% CI: 5% to 13.31%) and the false negative rate was 77.9% (95% CI: 71.88% to 83%). The precision of CXR was 42.3% (95% CI: 25.5% to 61.1%) and the accuracy was 74.1% (95% CI: 68.1% to 79.2%) (Table 3).

**Table 2:** Injury detection by modality (n = 239)

**Findings**	**CXR**	**TCT**	**Percent of injuries found on CXR**	**95% CI**
Pneumothorax	3	21	14.3%	4.14% to 35.48%
Rib fractures	13	33	39.4%	24.65% to 56.35%
Lung contusion	8	15	53.3%	30.11% to 75.20%
Sternal fractures	1	5	20%	2.03% to 64.04%

**Table 3:** Sensitivity and specificity of CXR (n = 239)

	**Thoracic injury**	**No thoracic injury**
**CXR**	(+) 11 (-) 47	(+) 15 (-) 166
**TCT**	58	181

(+) indicates a positive study; (-) indicates a negative study.

Sensitivity of CXR 19% (95% CI: 10.8% to 31%).

Specificity of CXR 91.7% (95% CI: 86.7% to 95%).

False positive rate 8.3% (95% CI: 5% to 13.31%).

False negative rate 81% (95% CI: 69% to 89.2%).

Precision of CXR 42.3% (95% CI: 25.5% to 61.1%).

Accuracy of CXR 74.1% (95% CI: 68.1% to 79.2%).

P-value 0.0299.

The hospital charge for a single view portable CXR performed in the trauma room at Saint Francis Hospital was $569.92. Adding radiologists’ fees increased the cost estimate to $594.92 per CXR. This yields an annual cost estimate of $142,185.88 using the portable device (Table 4).

**Table 4:** Cost of CXR per year

	**CXR**	**Professional fee**	**Total per CXR**	Total per year*
**Medicare**	$51.80	$9.46	$61.26	$14,641.14
**Activity based costing**	$103	$25.00	$128	$30,592
**Hospital portable**	$569.92	$25.00	$594.92	$142,185.88

* Based on 239 patients.

Direct hospital cost at Saint Francis Hospital was $103 per CXR, using the ABC method of estimation. Including the radiologist’s fees for interpreting the study, this cost increases to $128 per CXR, which brings the annual cost to $30,592 (Table 4).

Medicare reimbursement to the hospital for a single view CXR is $51.80. This does not include the professional fee for the radiologist’s interpretation, which is $9.46. Therefore, the total Medicare reimbursement for a CXR completed in the trauma room, including the contribution from the patient or the patient’s co-insurance and the radiologist’s fee, is $61.26 per CXR. This amounts to a total annual Medicare reimbursement for our series of CXRs of $14,641.14 (Table 4).

## Discussion

CXR is used in the early assessment of trauma patients to screen for significant thoracic injuries that require immediate intervention before leaving the trauma room. A good screening test is defined as being highly sensitive and moderately specific; in short, it should be a good tool for ruling out injury. In our study, CXR was compared with TCT scan as the gold standard. The sensitivity (true positive rate) of CXR was calculated to be 19% (95% CI: 10.8% to 31%) and the specificity (true negative rate) was 91.7% (95% CI: 86.7% to 95%). These numbers suggest that CXR is not, in fact, a good screening tool for thoracic injury in blunt trauma patients. CXR has a positive predictive value of 42.3% (95% CI: 25.52% to 61.08%), which is the probability that a positive finding on CXR reflects a true positive finding. The negative predictive value of CXR is 77.9% (95% CI: 71.88% to 83%), which reflects the probability that a negative finding on CXR reflects a true negative finding. The precision of CXR was 42.3% (95% CI: 25.5% to 61.1%), which reflects the reproducibility of a test result. The overall accuracy was 74.1% (95% CI: 68.1% to 79.2%).

This study supports previous findings in the literature which clearly demonstrate that TCT is superior to CXR in identifying thoracic injury in blunt trauma patients [2-4,11-17,20,22-25]. There remains, however, a great deal of controversy surrounding the discussion of whether or not TCT should be used routinely as an adjunct to the primary survey in blunt trauma patients. In a study from 1994, Marts et al. conclude that TCT is more sensitive and specific in detecting parenchymal and pleural injuries than CXR, but that the majority of injuries identified by CT alone are minor [14]. The standard protocol for TCT in this study was 1cm cuts from the apices of the lungs to the diaphragm, with contrast, and including both mediastinal and lung windows. Our protocol for TCT in trauma, as mentioned above, includes 5 mm cuts through the thorax with 3 mm reconstruction of the thoracic spine. The CT scanner at SFH also provides many windows for viewing the images, including pulmonary, abdomen, and bone. In Marts’ study, TCT was found to be inferior to CXR in the evaluation of bony trauma, however, no bone window was available for these TCTs. With the evolution of technology, Marts’ study and others like it may have become obsolete.

A prospective cohort study from 2000 by Guerrero-Lopez and colleges suggests that, while TCT detects more injuries than CXR and induces therapy changes in a considerable number of patients, this does not translate into improved clinical outcomes [19]. The outcomes measured in this study were ICU length of stay, ICU mortality, and time on mechanical ventilation. While these are valid metrics for evaluating short-term improvements in patient care, this paper fails to address the long-term implications of missed injuries, including issues of pain management, residual lung dysfunction, time to return to work, and psychological affects. Additional prospective studies are needed to address these important questions. Additionally, in this study the mean duration of all CT explorations was 32.43 ± 13.31 minutes, with the upper limit being a full 60 minutes in the CT scanner [19]. This is significantly longer than current technology allows, and represents outdated information regarding the speed of modern scanners. These data only highlight the improvements in technology since this study was published.

Cost is an ever more important consideration in medicine. An analysis of the cost and benefit of pelvic x-rays in the evaluation of blunt trauma by Feeney, et al. in 2011 suggests that there is a potential for significant cost savings if pelvic x-rays can be deferred in hemodynamically normal patients who will be evaluated by CT scan [26]. A similarly designed study by Wisbach et al. from 2007 explores the utility of CXR in the evaluation of blunt trauma patients [16]. They suggest that in hemodynamically normal blunt trauma patients with a normal physical exam, CXR appears to be unnecessary. A total of 487 patients fit this description, and they estimate the cost of obtaining these CXRs as $8,830, approximately $18.13 per CXR. There is no discussion of the basis for the per x-ray charge figure, and based on our data this is a gross underestimate of the cost of obtaining a CXR.

We used three methods of cost analysis as described above. ABC was used to estimate the direct hospital costs associated with obtaining a single anterior-posterior CXR. This estimate accounts for the cost of equipment, a technician to perform the test, hospital support staff including the presence of a nurse, as well as the radiologist’s fee for interpreting the study, and other factors. This number is often an underestimation, since it fails to take into account such factors as equipment maintenance and replacement, lost productivity, and the extremes of patient care often required in the case of trauma patients with multiple injuries. Using ABC our estimated cost in 2010 for obtaining CXRs in patients who also received TCT was $30,592, including radiologist fees.

Using hospital charges as a surrogate for cost, on the other hand, is generally an over-estimate of true cost. The usual and customary charges include a profit margin for the hospital in addition to the other costs associated with performing the test. Based hospital charges for CXR performed in the trauma room at SFH and including radiologists’ fees, this yields a cost estimate $142,185.88 per year, using a portable device.

We also used Medicare reimbursement to estimate the cost of obtaining a CXR in the trauma room. This modality tends to underestimate the actual cost, since the reimbursement is less that the usual and customary fees, and does not include reimbursement for many overhead expenses. The profit margin for hospitals is significantly smaller and is designed to only partially cover the consumables and labor costs associated with performing the test. In addition, this reimbursement does not take into account the lost productivity, equipment maintenance, and labor costs associated with performing the test. The Medicare reimbursement to SFH for a single view CXR, including radiologist fees, amounted to $61.26 per study, representing an annual reimbursement of $14,641.14.

This study has several limitations. Firstly, this is a relatively small sample size; only 239 patients met inclusion and exclusion criteria from 1910 initially seen in the trauma room. SFH is a moderate sized Level II trauma center. At larger institutions with higher patient volumes, the potential cost savings could be even larger. Also, in database studies there is always the possibility that errors occurred in the initial data query and that patients were missed who should otherwise have been included. There is no reason to suspect this is the case, however it is a possibility worth mentioning as it could theoretically affect our data collection. In the case of trauma patients, they could possibly be overlooked if they were evaluated in places other than the two trauma rooms. We have no reason to think that this actually happened in the collection of these data, but it should be mentioned as a possible confounder. Also, all trauma patients receive a new medical record number when they are evaluated in the trauma rooms, even if they have a previous medical record number from being seen at the hospital before. Once it is determined that they have a preexisting record, the two records are merged. It is possible that in the process, records or imaging studies may be improperly merged, and therefore not included in these data. We found no instances of this in our review.

This review did not address any possible adverse reactions patients may have had from receiving a TCT scan, including dye reactions and radiation exposure. Possible unfavorable outcomes associated with obtaining a CXR in the trauma room were not explored either. However, no adverse reactions or unfavorable outcomes were documented from either CXR or TCT in the 239 patients included in our study.

Most importantly, it was not possible to control for the fact that the radiologists reading the CXR and TCT were unblinded to the results of the other test. This would be of particular importance if the TCT identified multiple injuries and the CXR was re-visited to look for those injuries identified by TCT. There were numerous addenda to imaging studies included in this review, but it was unclear what prompted these addenda. Such biases must be taken into consideration for future studies. Possibilities for future research include a large, multi-center trial to better compare these imaging modalities and to better account for selection and radiologist bias.

Finally there is the question of the indication for the CT scans. Most times, the indication for CT is pain or tenderness on physical exam. However, infrequently, the indication for TCT is listed in the medical record as widened mediastinum on CXR (4 patients, 1.7% (95% CI 0.5% to 4.4%). Considering all of those patients, 3 patients (75%, (95% CI 28.9% to 96.6%)) proceeded to TCT and had a negative study. One patient (0.4%; (95% CI <0.01% to 2.6%)) was found to have an isolated rib fracture (single rib) that was not identified on physical exam, and also showed a pulmonary contusion in close proximity to the isolated rib fracture. It is possible that the CXR, therefore, in these 4 cases (1.7% of patients; (95% CI 0.5% to 4.4%)) led to the TCT, which in turn led to the serendipitous diagnosis of the actual injury in one patient (0.4%; (95% CI <0.01% to 2.6%)). This population of patients, however, by meeting the inclusion and exclusion criteria, was entirely awake and alert, and was fully examinable at the time they were evaluated. None of those injuries was clinically significant on chart review, however, and two were discharged from the emergency department. Still, further studies will be needed to fully elucidate the effect of eliminating the CXR on the missed injury rate, and that is a limitation of this study.

## Conclusion

CXR is an important adjunct to the primary survey for many trauma patients. In the case of penetrating trauma, particularly gunshot wounds, CXRs can provide vital data about trajectory and immediately life-threatening injuries in a matter of minutes. In patients with multiple injuries who are hemodynamically unstable, CXRs can often help elucidate the cause quickly and efficiently without leaving the trauma room. It remains a valuable tool in the armamentarium of the trauma surgeon.

However, we examined a small subset of patients, characterized by blunt mechanism, hemodynamic normality, respiratory normality, who are awake, alert and reliably examined, and who will proceed to TCT for abnormalities on history and physical exam, regardless of the CXR findings. In that cohort, we suggest that the routine CXR be omitted. This would eliminate an unnecessary test that in this population does not add useful diagnostic information and incurs significant cost as well as unnecessary radiation exposure.

Overall, the estimated annual cost savings if these 239 CXRs had not been done ranges from $14,641.14 to as much as $142,185.88. The true estimate is likely closer to the upper limit of this range. In addition, SFH is a Level II trauma center with a moderate volume of patients. The potential annual cost savings at a larger institution with a higher volume of patients would likely be considerably higher than our estimate.

## Competing interests

All authors declare that they have no financial or non-financial competing interests.

## Authors’ contributions

KZ participated in research design, data collection, data analysis, and manuscript preparation. JF participated in research design, data collection, data analysis, and manuscript preparation. CD participated in research design and data collection and manuscript preparation. DS participated in research design, data collection, and data analysis. WM participated in research design, data analysis, and manuscript preparation. MT participated in research design, data analysis, and manuscript preparation. All authors read and approved the final manuscripts.

## References

[B1] National Safety Council Report on Injuries in America 2009 Available: http://www.east.org/resources/treatment-guidelines/cervical-spine-injuries-following-trauma. Accessed: October 14, 2010

[B2] Trupka A Waydhas C Hallfeldt K Value of thoracic computed tomography in the first assessment of severely injured patients with blunt chest trauma: results of a prospective study J Trauma 2007 43 3 405 412 9314300 10.1097/00005373-199709000-00003

[B3] Peters S Nicolas V Heyer CM Multidetector computed tomography-spectrum of blunt chest wall and lung injuries in polytraumatized patients Clinical Rad 2010 65 333 338 10.1016/j.crad.2009.12.008 20338402

[B4] Deunk J Brink M Dekker HM Routine versus selective Multidetector-Row Computed Tomography (MDCT) in blunt trauma patients: level of agreement on the influence of additional findings on management J Trauma 2009 67 5 1080 1086 10.1097/TA.0b013e318189371d 19901671

[B5] Keough V Pudelek B Blunt chest trauma: review of selected pulmonary injuries focusing on pulmonary contusion AACN Clinical Issues 2001 12 2 270 281 10.1097/00044067-200105000-00010 11759554

[B6] Magret M Lung trauma Clinical Pulm Med 2010 17 2 75 81 10.1097/CPM.0b013e3181d269aa

[B7] American College of Surgeons Advanced Trauma Life Support for Doctors, Student Course Manual 2004 7 Chicago, IL: ACS

[B8] Eastern Association for the Surgery of Trauma Practice Management Guidelines Committee Identifying Cervical Spine Injuries Following Trauma 2009 Available: http://www.nsc.org/Documents/Injury_Facts/Injury_Facts_2011_w.pdf. Accessed: April 27, 2011 10.1097/TA.0b013e3181ae583b 19741415

[B9] Duane TM Deehert T Wolfe LG Clinical examination is superior to plain films to diagnose pelvic fractures compared to CT Am Surg 2008 74 476 80 18556988

[B10] Duane TM Tan BB Golay D Blunt trauma and the role of routine pelvic radiographs: a prospective analysis J Trauma 2002 Sep 53 3 463 8 10.1097/00005373-200209000-00011 12352481

[B11] Exadaktylos AK Sclabas G Schmid SW Do We really need routine computed tomographic scanning in the primary evaluation of blunt chest trauma in patients with “normal” chest radiograph? J Trauma 2001 51 1173 1176 10.1097/00005373-200112000-00025 11740271

[B12] Kea B Rodriguez RM Fortman J Do chest radiography miss significant intrathoracic injury in blunt trauma patients? Annals of Emerg Med 2010 53 suppl 3 S102

[B13] Blostein PA Hodgman CG Computed tomography of the chest in blunt thoracic trauma: results of a prospective study J Trauma 1997 43 1 13 18 10.1097/00005373-199707000-00006 9253901

[B14] Marts B Durham R Shapiro M Computed tomography in the diagnosis of blunt thoracic injury Amer J of Surgery 1994 168 6 688 692 10.1016/S0002-9610(05)80146-1 7978020

[B15] Guerrero-López F Vázquez-Mata G Alcázar-Romero PP Evaluation of the utility of computed tomography in the initial assessment of the critical care patient with chest trauma Crit Care Med 2000 May 28 5 1370 5 10.1097/00003246-200005000-00018 10834680

[B16] Wisbach GG Sise MJ Sack DI What is the role of chest X-ray in the initial assessment of stable trauma patients? J Trauma 2007 Jan 62 1 74 8 discussion 78–9 10.1097/01.ta.0000251422.53368.a3 17215736

[B17] Elmali M Baydin A Nural MS Lung parenchymal injury and its frequency in blunt thoracic trauma: the diagnostic value of chest radiography and thoracic CT Diagn Interv Radiol 2007 Dec 13 4 179 82 18092287

[B18] Bokhari F Brakenridge S Nagy K Prospective evaluation of the sensitivity of physical examination in chest trauma J Trauma 2002 53 1135 1138 10.1097/00005373-200212000-00017 12478040

[B19] Sears BW Luchette FA Esposito TJ Old fashion clinical judgment in the era of protocols: is mandatory chest X-ray necessary in injured patients? J Trauma 2005 Aug 59 2 324 30 discussion 330–2 10.1097/01.ta.0000179450.01434.90 16294071

[B20] Omert L Yeaney WW Protetch J Efficacy of thoracic computerized tomography in blunt chest trauma Am Surg 2001 Jul 67 7 660 4 11450784

[B21] Hollingworth W Radiology cost and outcomes studies: standard practice and emerging methods Am J Roentgenol 185 4 833 16177396 10.2214/AJR.04.1780

[B22] Neff MA Monk JS Jr Peters K Nikhilesh A Detection of occult pneumothoraces on abdominal computed tomographic scans in trauma patients J Trauma 2000 Aug 49 2 281 5 10.1097/00005373-200008000-00015 10963540

[B23] Brasel KJ Stafford RE Weigelt JA Treatment of occult pneumothoraces from blunt trauma J Trauma 1999 Jun 46 6 987 90 discussion 990–1 10.1097/00005373-199906000-00001 10372613

[B24] Wolfman NT Myers WS Glauser SJ Validity of CT classification on management of occult pneumothorax: a prospective study AJR Am J Roentgenol 1998 Nov 171 5 1317 20 10.2214/ajr.171.5.9798871 9798871

[B25] Hill SL Edmisten T Holtzman G Wright A The occult pneumothorax: an increasing diagnostic entity in trauma Am Surg 1999 Mar 65 3 254 8 10075304

[B26] Feeney J Jayaraman V Luk S Retrospective review of the costs of routine pelvic X-rays in a trauma setting Am Surg 2011 Mar 77 3 337 41 21375847

